# SBSI: an extensible distributed software infrastructure for parameter estimation in systems biology

**DOI:** 10.1093/bioinformatics/btt023

**Published:** 2013-01-17

**Authors:** Richard Adams, Allan Clark, Azusa Yamaguchi, Neil Hanlon, Nikos Tsorman, Shakir Ali, Galina Lebedeva, Alexey Goltsov, Anatoly Sorokin, Ozgur E. Akman, Carl Troein, Andrew J. Millar, Igor Goryanin, Stephen Gilmore

**Affiliations:** ^1^SynthSys Edinburgh, King's Buildings, University of Edinburgh, Edinburgh, UK, ^2^School of Informatics, Informatics Forum, University of Edinburgh, Edinburgh, UK, ^3^BSkyB, Watermark House, Livingston, Scotland, ^4^The Systems Biology Institute, Falcon Building 5F, 5-6-9 Shirokanedai, Tokyo 108-0071, Japan, ^5^Horizon Digital Economy Research, University of Nottingham, Nottingham, UK, ^6^University of Abertay Dundee, Dundee, UK, ^7^Institute of Cell Biophysics RAS, Institutskaya st., 3, Pushchino, Russia, ^8^Centre for Systems, Dynamics and Control, University of Exeter, UK and ^9^Computational Biology and Biological Physics, Lund University, Sölvegatan 14A, Lund, Sweden

## Abstract

**Summary:** Complex computational experiments in Systems Biology, such as fitting model parameters to experimental data, can be challenging to perform. Not only do they frequently require a high level of computational power, but the software needed to run the experiment needs to be usable by scientists with varying levels of computational expertise, and modellers need to be able to obtain up-to-date experimental data resources easily. We have developed a software suite, the Systems Biology Software Infrastructure (SBSI), to facilitate the parameter-fitting process. SBSI is a modular software suite composed of three major components: SBSINumerics, a high-performance library containing parallelized algorithms for performing parameter fitting; SBSIDispatcher, a middleware application to track experiments and submit jobs to back-end servers; and SBSIVisual, an extensible client application used to configure optimization experiments and view results. Furthermore, we have created a plugin infrastructure to enable project-specific modules to be easily installed. Plugin developers can take advantage of the existing user-interface and application framework to customize SBSI for their own uses, facilitated by SBSI’s use of standard data formats.

**Availability and implementation:** All SBSI binaries and source-code are freely available from http://sourceforge.net/projects/sbsi under an Apache 2 open-source license. The server-side SBSINumerics runs on any Unix-based operating system; both SBSIVisual and SBSIDispatcher are written in Java and are platform independent, allowing use on Windows, Linux and Mac OS X. The SBSI project website at http://www.sbsi.ed.ac.uk provides documentation and tutorials.

**Contact**: stg@inf.ed.ac.uk

**Supplementary information**: Supplementary data are available at *Bioinformatics* online.

## 1 THE SBSI SYSTEM

SBSI is a modular software system, consisting of three major software components: (i) SBSINumerics, containing core numerical global search algorithms; (ii) SBSIDispatcher, providing access to back-end HPC machines; and (iii) SBSIVisual, a client application that provides a user-friendly consistent user interface to the tools provided by the SBSINumerics framework. Supplementary Material S1 illustrates this architecture.

Each module is developed independently, and there are no dependencies between modules, facilitating re-use of code. Therefore each module may be installed independently depending on user requirements, ranging from simple command-line access to SBSINumerics, to the full stack of client application, webserver and back-end task server.

The provision of parallel execution for parameter optimization means that SBSI has the potential to scale to higher-dimensional modelling problems than serial codes such as PottersWheel (Maiwald and Timmer, 2008) and DOTcvpSB (Hirmajer *et al.*, 2009). In addition, unlike these optimizers, SBSI has no dependency on the MATLAB software.

SBSI offers a unique advantage over other parallel suites such as SBML-PET-MPI (Zi, 2011) in that SBSI provides an information privacy infrastructure that obfuscates the models and data that are sent to the HPC back-end. This ensures that the server receives the minimum information necessary to execute the computation, ensuring confidentiality of the model semantics. Communication between modules is secured through protocols such as https and ssh. Authentication details are encrypted using one-way hashing.

## 2 THE COMPONENTS OF SBSI

SBSINumerics is a C++ library that provides parallelized implementations of global optimization algorithms such as genetic algorithms, particle swarm and simulated annealing. SBSINumerics is optimized for performance: models are converted to C++ and compiled into the library before execution, and parallelization enables substantial speed-up of the search on multi-processor computers. Two objective functions are provided: a least-squares difference function to determine the goodness-of-fit of a simulation to experimental data, and a Fast Fourier Transform algorithm that identifies oscillatory models. It is also possible to link to external cost functions specific to an individual model.

SBSIDispatcher provides a secure REST-ful webservice API to access parameter fitting and simulation functionality on the SBSI servers. This middleware layer reduces coupling between client applications and the servers that run the jobs, allowing different HPCs or clusters to be utilized with little configuration required by the end-user. The web-service endpoint and documentation is https://sbsidev1.inf.ed.ac.uk:8083/sbsiservices/relations/list. This API enables applications other than SBSIVisual to use the SBSINumerics algorithms and SBSI servers. We developed a web application at https://sbsidev1.inf.ed.ac.uk:8083/sbsiservices/ that tracks job progress and accesses results. We developed the server application in Java using the industry-standard Spring MVC framework, and it can be deployed as a standard web application.

SBSIVisual is a client application to configure parameter optimization jobs, to link with external databases and to view the results of optimizations.

It provides a workspace where local copies of models, data and diagrams can be worked on, and provides validation services for these resources. Time series simulations can be performed using pre-installed ODE solvers from the Copasi distribution (Mendes *et al.*, 2009), the CVODE package (https://computation.llnl.gov/casc/sundials/main.html) and Dizzy (Ramsey *et al.*, 2005), but other simulators can be installed as plugins. A screenshot is shown in Supplementary Material S2.

One of the main purposes of SBSIVisual is to make it easier for end-users to configure optimization tasks, which can be quite complex. Such configuration includes the choice of which parameters to optimize and their constraints, the optimization algorithm and its parameters, and the choice of data sets and cost functions (which may need to be configured on a per-dataset basis). SBSIVisual uses a wizard dialogue to guide the user through this process, and stores the configuration as an SBML Annotation in the model file. This configuration populates the wizard on subsequent occasions, easing the configuration process further. Additionally, users may now exchange both parameter-fitting configurations and models.

SBSIVisual provides a report view of a parameter-fitting experiment, showing plots of cost function value versus search iteration, and overlays of simulation traces and experimental data. In addition, if the user elects to retain the full search history, a cobweb plot can be created to show how the search space has been explored. An example plot is shown in Supplementary Material S3.

SBSIVisual is written in Java using the Eclipse Rich Client Platform (RCP) as an application framework. This framework offers a great deal of user–interface support, as well as a robust and highly developed plugin system for adding new functionality. Our use of standard data formats such as the Systems Biology Markup Language [SBML, Hucka *et al.* (2003)] provides a stable basis for plugin development. Plugins can be downloaded and installed from an *update site* (http://www.sbsi.ed.ac.uk/update) with minimal user input. Plugins developed to date enable access to project-specific databases and editing support for standard data formats such as SBML and SED-ML (Adams *et al.*, 2012; Adams, 2012).

## 3 CONCLUSIONS

The main focus of SBSI is the task of parameter estimation, which by its nature is computationally intensive. Progress of long-running jobs can be monitored by observing cost function values, and downloading intermediate result files. At the end of a parameter fitting, the user can download the fitted model, together with simulation results generated from the model, and a full log of the explored search space. Since it is beyond our resources to provide computational time for everyone wishing to use SBSI, we have endeavoured to package the software in a form that can be easily installable on other servers. SBSIDispatcher, which acts as a task manager for remotely running jobs, is deployed as a regular Java web application, and only requires configuration of database access in order to be useable on other application servers.

## Supplementary Material

Supplementary Data
