# Supplementary material for: SBSI: an extensible distributed software infrastructure for parameter estimation in systems biology
Source: Bioinformatics. 2013 Jan 17;29(5):664–5. doi: 10.1093/bioinformatics/btt023 (PMC3582266; doi:10.1093/bioinformatics/btt023)
Supplement: Supplementary Data [file supp_29_5_664__index.html]

SBSI: An extensible distributed software infrastructure for parameter estimation in systems biology — SBSI: an extensible distributed software infrastructure for parameter estimation in systems biology — SBSI: an extensible distributed software infrastructure for parameter estimation in systems biology — Supplementary Data 

# SBSI: an extensible distributed software infrastructure for parameter estimation in systems biology

## Supplementary Data

files

**Files in this Data Supplement:**

- Supplementary Data - pdf file
