# Supplementary material for: SBSI: an extensible distributed software infrastructure for parameter estimation in systems biology
Source: Bioinformatics. 2013 Jan 17;29(5):664–5. doi: 10.1093/bioinformatics/btt023 (PMC3582266; doi:10.1093/bioinformatics/btt023)
Supplement: Supplementary Data [file supp_btt023_SuppMats.pdf]

SBSI - An extensible software infrastructure for Systems Biology modelling

Richard Adams [1], Allan Clark [1], Azusa Yamaguchi [5], Neil Hanlon [2], Nikos Tsorman [3], Shakir Ali [4], Galina Lebedeva [1], Alexey Goltsov [6], Anatoly Sorokin [7], Ozgur E. Akman [8], Carl Troein [9], Andrew J. Millar[1], Igor Goryanin[5], and Stephen Gilmore [1,5*]

[1]SynthSys Edinburgh, CH Waddington Building, King's Buildings, University of Edinburgh, Edinburgh,UK.

[2]BSkyB, Watermark House, Livingston, Scotland.

[3]The Systems Biology Institute, Falcon Building 5F, 5-6-9 Shirokanedai, Tokyo 108-0071 Japan.

[4]Horizon Digital Economy Research, University of Nottingham, Nottingham, UK.

[5]School of Informatics, Informatics Forum, University of Edinburgh, Edinburgh, UK.

[6]Centre for Research in Informatics and Systems Pathology (CRISP), University of Abertay Dundee, Dundee, UK.

[7]Institute of Cell Biophysics RAS, Institutskaya st., 3, Pushchino, Russia.

[8]Centre for Systems, Dynamics and Control, College of Engineering, Mathematics & Physical Sciences, University of Exeter, UK.

[9]Computational Biology and Biological Physics, Lund University, Sölvegatan 14A, Lund, Sweden.

[*]to whom correspondence should be addressed

Supplementary Materials

S1 - SBSI software architecture

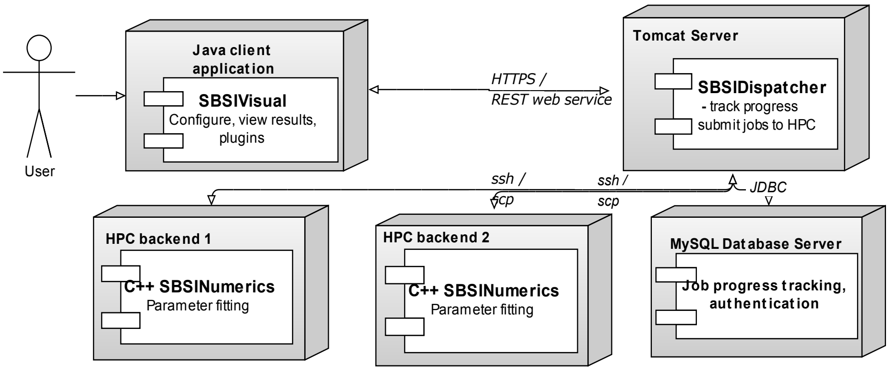

Figure 1: SBSI software components comprising a client application (SBSIVisual), a web service (SBSIDispatcher) and analytical modules (SBSINumerics) deployed on standard servers or HPC machines.

S2 - visualization of search results

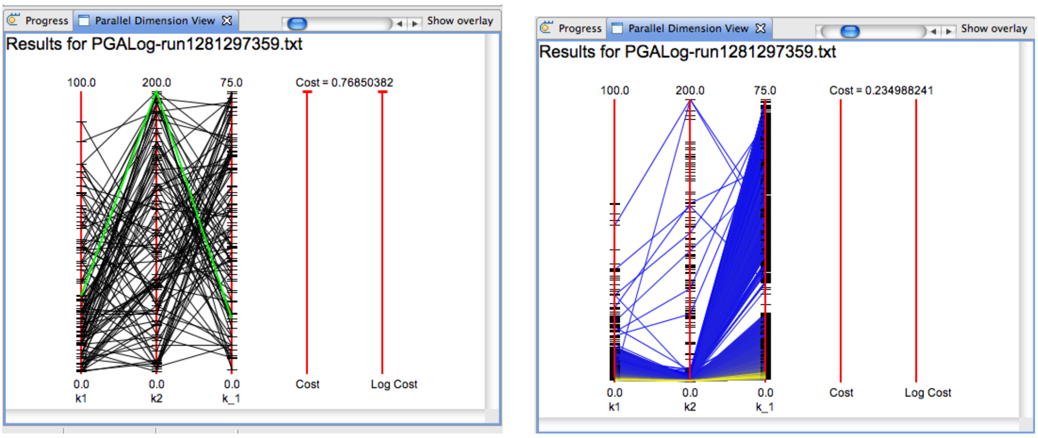

(a) (b)

Figure 2: Panel A) shows a cobweb or parallel dimension plot of a simple optimisation run. In this view, each red vertical line represents a parameter. A point on the line represents a value for that parameter. The range of values is limited by the constraints imposed on the search. The black lines connect the values of the parameters for a particular parameter set that has been evaluated during the optimization. So, each black line represents an individual in the population, in a single generation. The number of black lines is equal to the population size. The best individual for that generation is highlighted in green. In the example above, there are 100 black lines, because the population size was 100. Panel B) shows a superimposition of plots of the best individuals for each iteration of the search, colour coded from blue (poor fitting, high cost) to yellow (better fitting, low cost). This graph reveals that possible values for the parameter k_1 were fully explored within the parameter constraints, but values for k_2 were quite fixed at a low value. This could indicate a local minimum in the search space that the search algorithm was unable to escape from. This could then guide the user to alter the behaviour of the search algorithm to vary the parameter values more after each iteration, in order to escape from such a minimum.

S3 - the SBSIVisual application

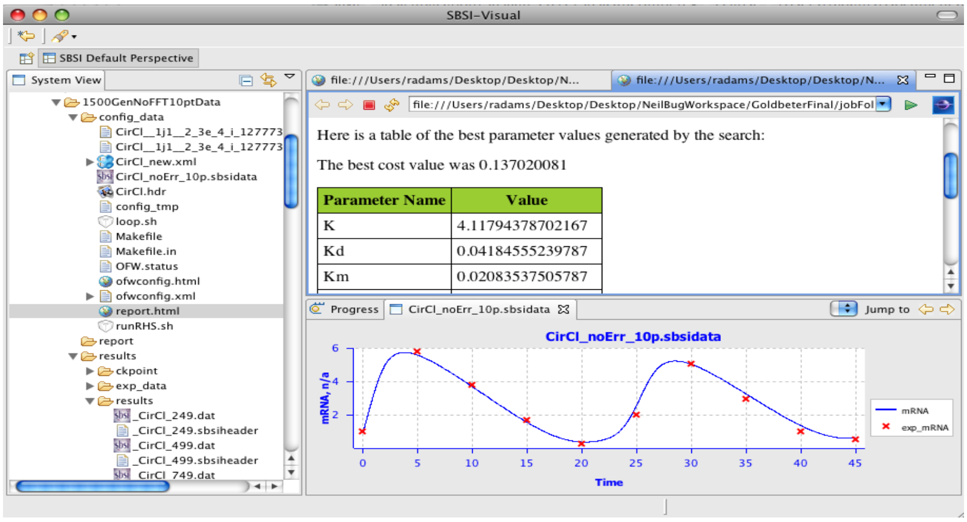

Figure 3: Screenshot of SBSIVisual showing workspace, editor pane, and views of optimisation results.
